# ERK inhibitor ASN007 effectively overcomes acquired resistance to EGFR inhibitor in non‐small cell lung cancer

**DOI:** 10.1007/s10637-021-01121-6

**Published:** 2022-01-01

**Authors:** Bo Mi Ku, Jae Yeong Heo, Jinchul Kim, Jong-Mu sun, Se-Hoon Lee, Jin Seok Ahn, Keunchil Park, Myung-Ju Ahn

**Affiliations:** 1 Research Institute for Future Medicine, Samsung Medical Center, Sungkyunkwan University School of Medicine, Seoul, Korea; 2 Division of Hematology and Oncology, Department of Medicine, Samsung Medical Center, Sungkyunkwan University School of Medicine, 81 Irwon-ro, Gangnam-gu, Seoul, 06351 Korea

**Keywords:** NSCLC, EGFR TKI, Acquired resistance, ERK inhibitor, Combination strategy

## Abstract

The emergence of acquired resistance limits the long-term efficacy of EGFR tyrosine kinase inhibitors (EGFR TKIs). Thus, development of effective strategies to overcome resistance to EGFR TKI is urgently needed. Multiple mechanisms to reactivate ERK signaling have been successfully demonstrated in acquired resistance models. We found that in EGFR mutant non-small cell lung cancer (NSCLC) patients, acquired resistance to EGFR TKIs was accompanied by increased activation of ERK. Increased ERK activation was also found in *in vitro* models of acquired EGFR TKI resistance. ASN007 is a potent selective ERK1/2 inhibitor with promising antitumor activity in cancers with *BRAF* and *RAS* mutations. ASN007 treatment impeded tumor cell growth and the cell cycle in EGFR TKI-resistant cells. In addition, combination treatment with ASN007 and EGFR TKIs significantly decreased the survival of resistant cells, enhanced induction of apoptosis, and effectively inhibited the growth of erlotinib-resistant xenografts, providing the preclinical rationale for testing combinations of ASN007 and EGFR TKIs in *EGFR*-mutated NSCLC patients. This study emphasizes the importance of targeting ERK signaling in maintaining the long-term benefits of EGFR TKIs by overcoming acquired resistance.

## Introduction

Although EGFR tyrosine kinase inhibitors (EGFR TKIs) significantly improve clinical outcomes in patients with non-small cell lung cancer (NSCLC) harboring *EGFR* mutations, such as exon 19 deletion and the L858R mutation, almost all mutant *EGFR*-positive NSCLC patients ultimately acquire resistance to EGFR TKIs after approximately 1–2 years [1]. Therefore, overcoming acquired resistance is still crucial to improving therapeutic efficacy. The most common acquired resistance mechanism is acquisition of another resistant mutation in *EGFR*, such as T790M and C797S. In addition, resistance to EGFR TKIs can also be driven by constitutive activation of the MEK-ERK pathway, as demonstrated previously [2–7].

MEK-ERK signaling is a key pathway downstream of EGFR and mediates EGFR-dependent regulation of cancer cell growth and survival. Previous studies have demonstrated that sustained ERK activation is involved in resistance to EGFR TKIs. Thus, targeting MEK-ERK signaling through either an MEK or ERK inhibitor can overcome acquired resistance to EGFR TKIs. Aberrant activation of ERK signaling was found in erlotinib-, gefitinib-, osimertinib-, and WZ4002-resistant NSCLC cells [2, 5–12]. Furthermore, in erlotinib- and gefitinib-resistant cells, the combination of EGFR TKI with an MEK inhibitor effectively inhibited tumor growth and impeded the development of resistance [8, 9]. Similarly, the combination of osimertinib with an MEK or ERK inhibitor synergistically induced cell death in osimertinib-resistant NSCLC cells [2, 7, 10–12]. MEK inhibition using trametinib combined with WZ4002 was shown to delay the emergence of acquired resistance to WZ4002 in NSCLC [4].

FOS-related antigen 1 (FRA1) is an ERK-dependent oncogenic transcription factor and a member of the AP-1 transcriptional factor superfamily. As ERK amplitude and duration both contribute to the induction and activation of FRA1, the expression and phosphorylation levels of FRA1 protein linearly reflect ERK activity [13, 14]. FRA1 is frequently upregulated in a wide variety of tumors and has important roles during consecutive stages of multistep tumor progression by promoting cell proliferation, inhibiting apoptosis and enhancing tumor angiogenesis. In addition, FRA1 may promote cancer progression by facilitating immune evasion through PD-L1 expression in high-risk, premalignant bronchial epithelial cells [14].

ERK is immediately downstream of MEK and is important to many cellular processes. ERK is responsible for phosphorylating a broad range of substrates involved in cell proliferation, differentiation, and survival. Selective ERK inhibitors have been developed and used in clinical trials for the treatment of a variety of cancers such as melanoma, pancreatic cancer, and NSCLC [15]. However, given the negative feedback upregulation of MEK and limited activity of ERK inhibitors alone, current strategies include combination treatment with an MEK inhibitor to impede ERK activation. Furthermore, selective ERK inhibitors may be a promising strategy for minimizing toxicity and enhancing activity.

ASN007 is an oral ERK1/2 inhibitor; an open-label, dose-escalation phase I study of ASN007 began in January 2018 and is still ongoing. However, to date, little is known about function of ASN007 in preclinical models. Here, we investigated whether ASN007 alone or combination can overcome acquired resistance to EGFR TKIs in NSCLC.

## Materials and methods

### Patient tissue samples and immunohistochemistry

 Patients treated with EGFR TKIs (erlotinib, gefitinib, and afatinib) at Samsung Medical Center were retrospectively identified based on baseline and post-progression FFPE tissue availability. All procedures involving tumor specimens were reviewed and approved by the Institutional Review Board (IRB) of Samsung Medical Center (No. SMC 2010-04-039, 2011-10-054, 2013-08-113, and 2013-10-112), and written informed consent was provided by patients; in some cases, a waiver of consent was obtained. Paired tissue sections were obtained form 34 NSCLC patients and used for p-ERK1/2 staining. Immunohistochemistry was performed on 4-µm sections of formalin-fixed paraffin-embedded samples. Following deparaffinization and rehydration of the slides, antigen retrieval was performed using citrate buffer (pH 6.0). After endogenous peroxidase activity was blocked with 3 % hydrogen peroxide, sections were incubated with primary antibody for p-ERK1/2 (1:300, #4376; Cell Signaling Technology). Sections were further processed with horseradish peroxidase-conjugated secondary antibody and then developed with 3,3-diaminobenzidine. Finally, the slides were counterstained with hematoxylin. Slides were scanned with the Scanscope XT (Aperio Leica BioSystems) and analyzed with ImageJ software.

### Cell cultures and reagents

As previously described [2], an erlotinib-resistant cell line (PC9/ER) and osimertinib-resistant cell lines (PC9/OR and H1975/OR) have been established in our laboratory. Resistant cells contained original *EGFR* mutations, but had no additional *EGFR* mutations such as T790M or C797S. Cells were cultured in RPMI-1640 medium supplemented with 10 % FBS, penicillin (100 U/ml), and streptomycin (100 µg/ml) at 37 °C in a humidified atmosphere containing 5 % CO_2_. ASN007 was provided by Asana BioSciences (Bridgewater, New Jersey). Erlotinib and osimertinib were obtained from Selleckchem. All drugs were dissolved in dimethyl sulfoxide (DMSO) at a 10 mM concentration and stored at -20 °C until further use.

### Cell viability assay and colony formation assay

Cell viability was determined using a Cell Counting Kit (Dojindo Molecular Technologies) according to the manufacturer’s instructions. Colony formation assay was used to measure long-term cell viability, Briefly, cells were seeded in 6-well plates and allowed to attach overnight. Following the indicated treatment, drug containing medium was changed every 3 days. After 10–14 days, the colonies were fixed and stained with crystal violet.

###  Western blotting and antibodies

Cells were lysed on ice in lysis buffer (50 mM Tris (pH 8.0), 150 mM NaCl, 1 % NP-40) supplemented with a protease and phosphatase inhibitor cocktail (Sigma). Equal amounts of protein were then subjected to SDS-PAGE and transferred to polyvinylidene difluoride membranes. After blocking in 5 % skim milk, membranes were incubated with primary antibodies to p-ERK1/2 (Thr202/Tyr204). ERK1/2, p-FRA1(Ser265), FRA1, p-p90RSK (S380), RSK1, FoxM1, Aurora A, PLK1, Cyclin D1, CyclinB1, and PARP (Cell Signaling Technology) and β-actin (Santa Cruz Biotechnology). Membranes were then incubated with the appropriate second antibodies and developed using ECL (Pierce).

### Cell cycle analysis

Cell cycle analysis was performed after 24 h of treatment. Cells were fixed with ice-cold 70 % ethanol, stained with propidium iodide, and analyzed by flow cytometry (BD Biosciences).

### Xenograft studies

 The protocol involving all procedures about animals was reviewed and approved by the Institutional Animal Care and Use Committee (IACUC) at Samsung Biomedical Research Institute (SBRI). They are in accordance with the relevant national and international guidelines. Six-week-old BALB/c female nude mice were injected subcutaneously with PC9/ER cells. When tumor size reached approximately 100 mm^3^, mice were randomly assigned to groups of 4–6 mice each. ASN007 was dissolved in 0.5 % methyl cellulose containing 0.1 % Tween-80 and given orally. To evaluated the synergistic effect of EGFR TKI and ASN007, each group of mice was dosed with vehicle, ASN007 (50 mg/kg/d), erlotinib (25 mg/kg/d), or a combination of both by oral gavage 5 days per week. Tumor volumes were determined using calipers and calculated using the following formula: V = (L x W^2^)/2 (L, length; W, width). Mice were monitored daily with humane endpoints including a tumor greater than 1,500 mm^3^, weight loss of over 15 % of body mass, vomiting or skin problems, or inability to ambulate or rise for food and water. These humane endpoints were not observed in any mouse. All efforts were made to alleviate suffering. Mice were euthanized by CO_2_ inhalation at the end of the experiment. Tissues obtained after sacrifice were used for molecular analysis and Ki-67 (1:200, #9027; Cell Signaling Technology) staining.

### Statistical analysis

Data are presented as mean ± SEM. Statistical analyses were carried out using GraphPad Prism (GraphPad software). Correlation analysis was conducted using Pearson’s correlation. Statistical evaluation was performed with a two-tailed Student’s t-test, and *P* values < 0.05 were considered statistically significant.

## Results

### Acquired resistance to EGFR TKI is related to increased ERK activation in NSCLC tumor tissue

**Fig. 1:**
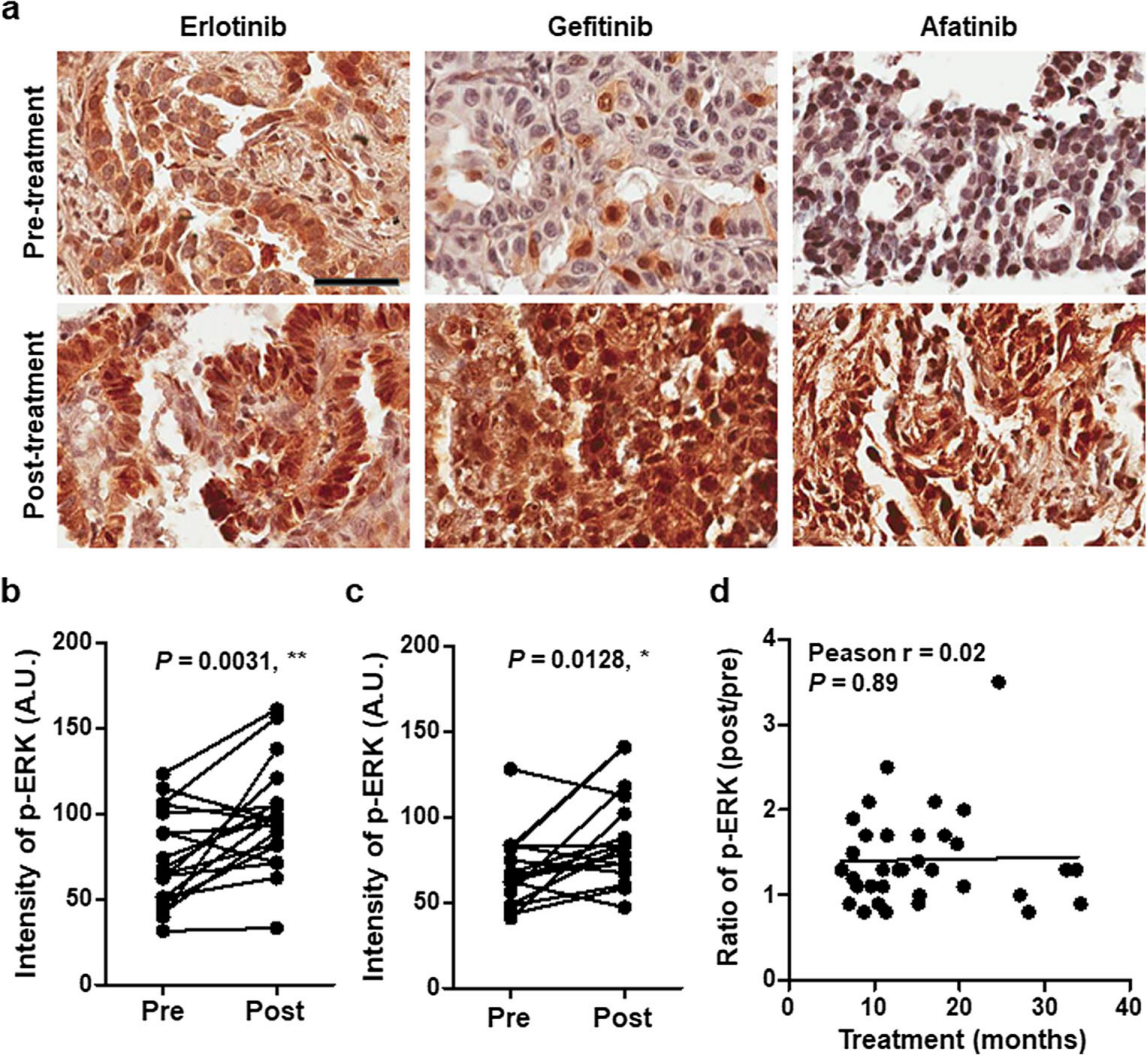
Phosphorylation of ERK is increased in NSCLC tumor samples at disease progression after EGFR TKI treatment. **a** Representative images of p-ERK1/2 staining from paired tumor samples treated with erlotinib, gefitinib, and afatinib. Top, tumor biopsy before EGFR TKI treatment. Bottom, tumor biopsy after EGFR TKI treatment at disease progression. Scale bar: 50 μm **b** Quantification of p-ERK1/2 staining score in the first-generation EGFR TKI (erlotinib and gefitinib) group (n = 18). **c** Quantification of p-ERK1/2 staining score in the second-generation EGFR TKI (afatinib) group (n = 16). **d** Correlation between the fold increase in p-ERK staining and treatment period. *, *P* < 0.05; **, *P* < 0.01

To determine whether increased ERK activation is related to *EGFR*-independent bypass resistance in NSCLC, we retrospectively analyzed 34 paired (pre- and post-treatment) biopsy samples from EGFR TKI treated patients. The treatment regimen in this cohort was erlotinib in 4 patients (11.8 %), gefitinib in 14 patients (41.2 %), and afatinib in 16 patients (47.1 %). Two patients (one treated with erlotinib and another with afatinib) received osimertinib as second-line treatment before re-biopsy. Consistent with a previous report [9], 26 (76.5 %) tumor biopsies collected at disease progression after EGFR TKIs treatment showed high level ERK phosphorylation on immunohistochemistry (Fig. 1a). Activated ERK level was significantly higher in post-treatment biopsy compared with baseline regardless of EGFR TKI regimen (Fig. 1b and c), suggesting that increased ERK activation may be associated with acquired resistance to EGFR TKI in EGFR-mutant NSCLC. However, the treatment period of EGFR TKI showed no correlation with ERK activation levels (Fig. 1d).

### ERK inhibitor ASN007 inhibits cell proliferation in EGFR TKI-resistant NSCLC cells

**Fig. 2:**
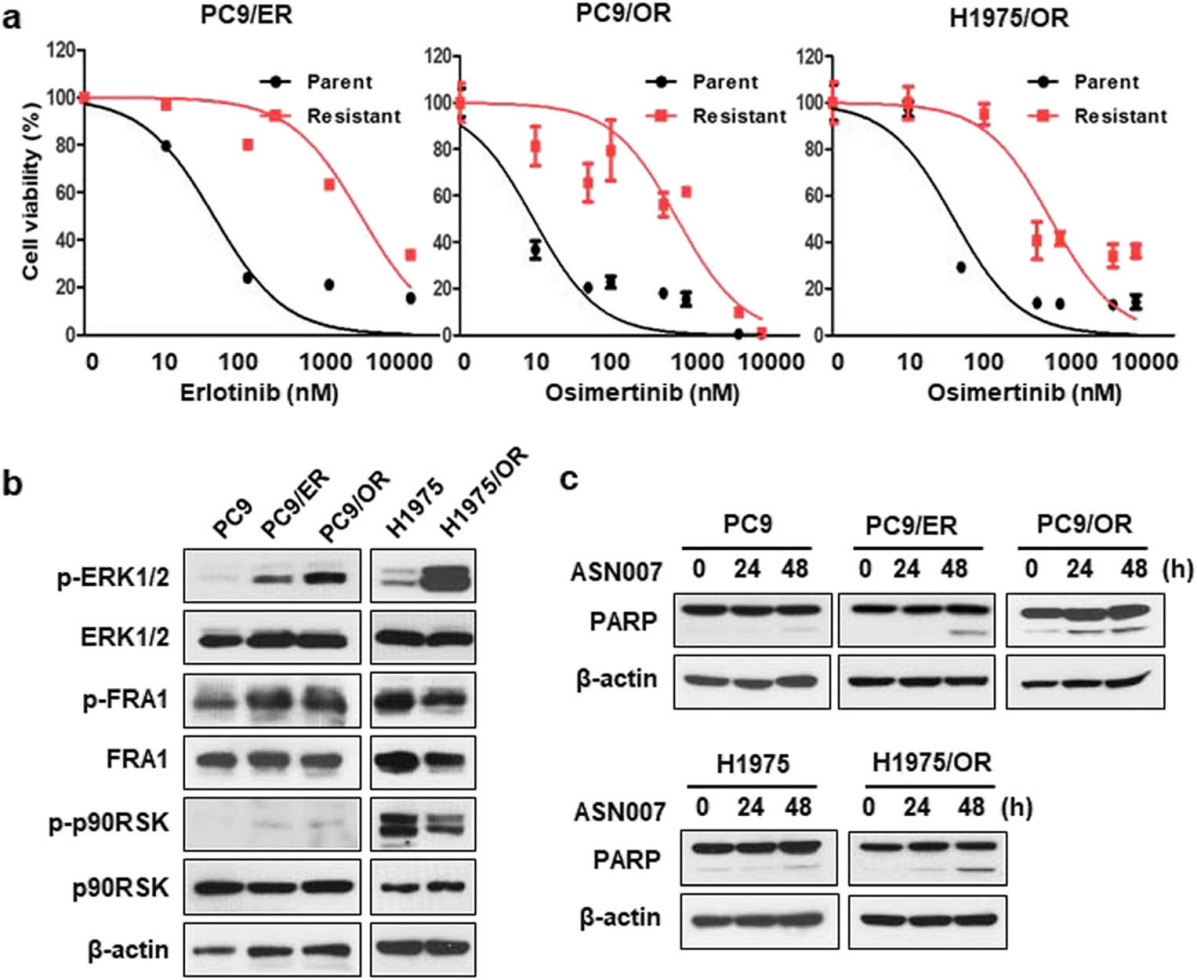
ERK inhibitor ASN007 induces cell death in EGFR TKI-resistant NSCLC cell lines. **a** Cell viability of erlotinib-resistant (PC9/ER) and osimertinib-resistant (PC9/OR and H1975/OR) cells after EGFR TKI treatment. **b** Basal protein expression levels in EGFR TKI-resistant NSCLC cell lines. **c** Assessment of apoptosis on ASN007 (500 nM) treatment. β-actin was used as a loading control

To evaluate the impact of ERK activation in acquired resistance to EGFR TKI, we examined the activation level of ERK and ERK substrates in both parental (PC9 and H1975) and EGFR TKI-resistant cells (erlotinib-resistance: PC9/ER, osimertinib-resistance: PC9/OR and H1975/OR) (Fig. 2a). Each resistance cell line harbors genomic alterations involved in ERK activation (PC9/ER: *NRAS* Q61H, PC9/OR: *HRAS* G13R, H1975/OR: *MAP2K1* K57E). The resistant cells showed increased ERK phosphorylation compared to parental cells (Fig. 2b). Unlike evident phosphorylation of FRA1, p90RSK phosphorylation was rarely detected in PC9, PC9/ER, and PC9/OR cells regardless of ERK phosphorylation status. To determine whether the survival of the resistant clones was the result of increased ERK activation, we treated ERK inhibitor, ASN007 to parent and resistant cells. ASN007 induced apoptotic cell death, as indicated by cleaved PARP and PAPR cleavage was more prominent in EGFR TKI-resistant cells than parent cells (Fig. 2c).

### ASN007 induces cell cycle arrest through FRA1 regulation

**Fig. 3:**
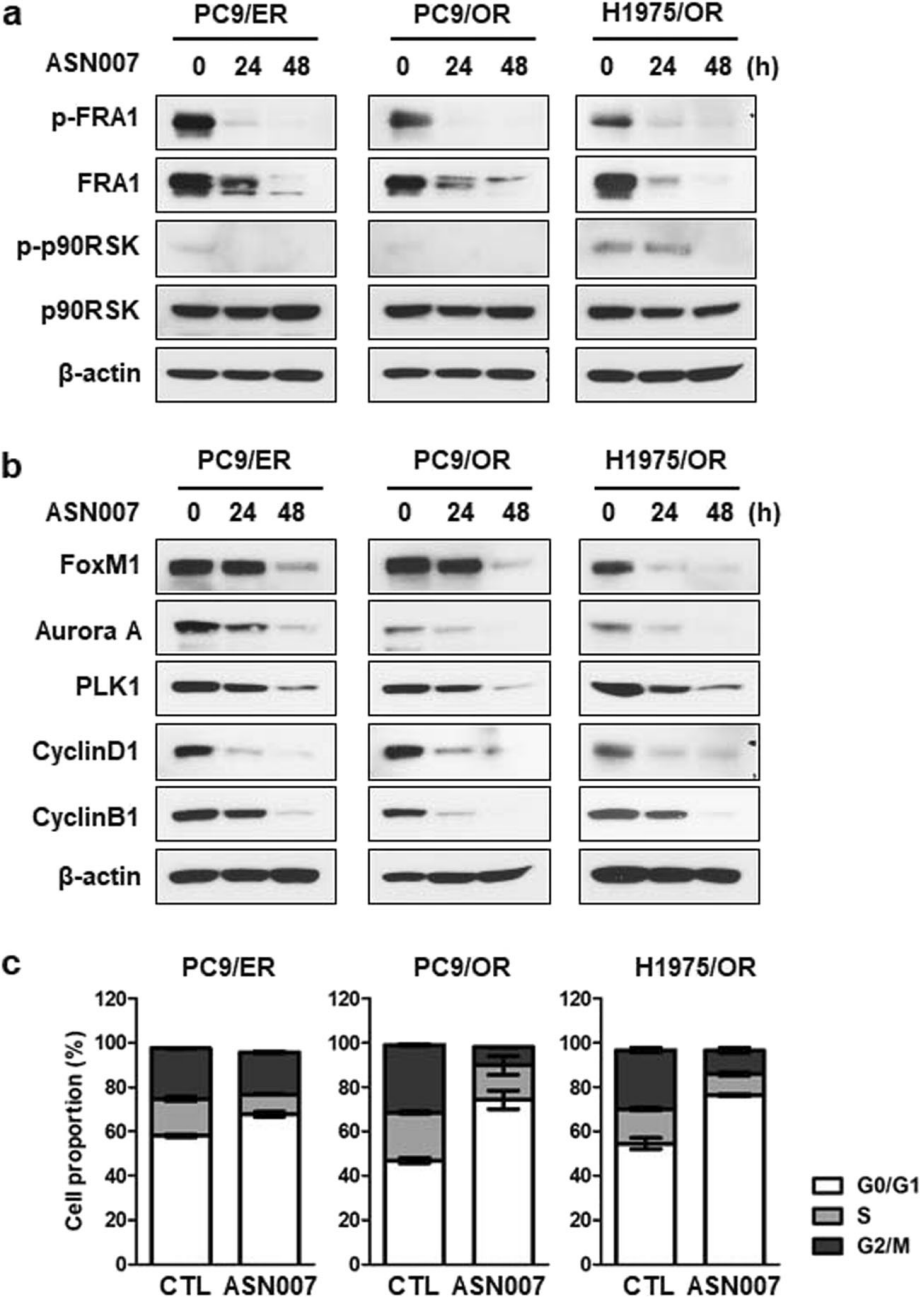
ASN007 inhibits FRA1 phosphorylation and mitosis-related protein expression in the ERK-downstream pathway. **a** Analysis of FRA1 and p90RSK, ERK-downstream signaling, and activation upon ASN007 (500 nM) treatment. **b** Analysis of mitosis-related protein expression after ASN007 (500 nM) treatment. β-actin was used as a loading control. **c** Cell cycle analysis after 24 h of ASN007 (500 nM) treatment (n = 3)

To identify the underlying mechanism by which ASN007 induces apoptosis in the EGFR TKI-resistant cells, we examined ERK substrate FRA1 and p90RSK. The treatment of ASN007 resulted in decreased FRA1 phosphorylation and protein expression in a time-dependent manner in all EGFR TKI-resistant cells (Fig. 3a), suggesting that FRA1 is more prominent mediator for ERK signaling than p90RSK in our model system. In previous reports, FRA1 directly regulated the expression of cell cycle-related protein, thereby promoting cell proliferation [16, 17]. In line with FRA1 inhibition, ASN007 decreased expression of cell-cycle-related proteins such as FoxM1, Aurora A, PLK1, Cyclin D1, and Cyclin B1 in all EGFR TKI-resistant cells (Fig. 3b). The effects of ASN007 on the cell cycle of EGFR TKI-resistant cells were analyzed using flow cytometry. The cell cycle was arrested at the G_0_/G_1_ phase in PC9/ER, PC9/OR, and H1975/OR cells following treatment with 500 nM ASN007 for 24 h (Fig. 3c).

### ASN007 can effectively overcome ERK-driven acquired resistance to EGFR TKI

**Fig. 4:**
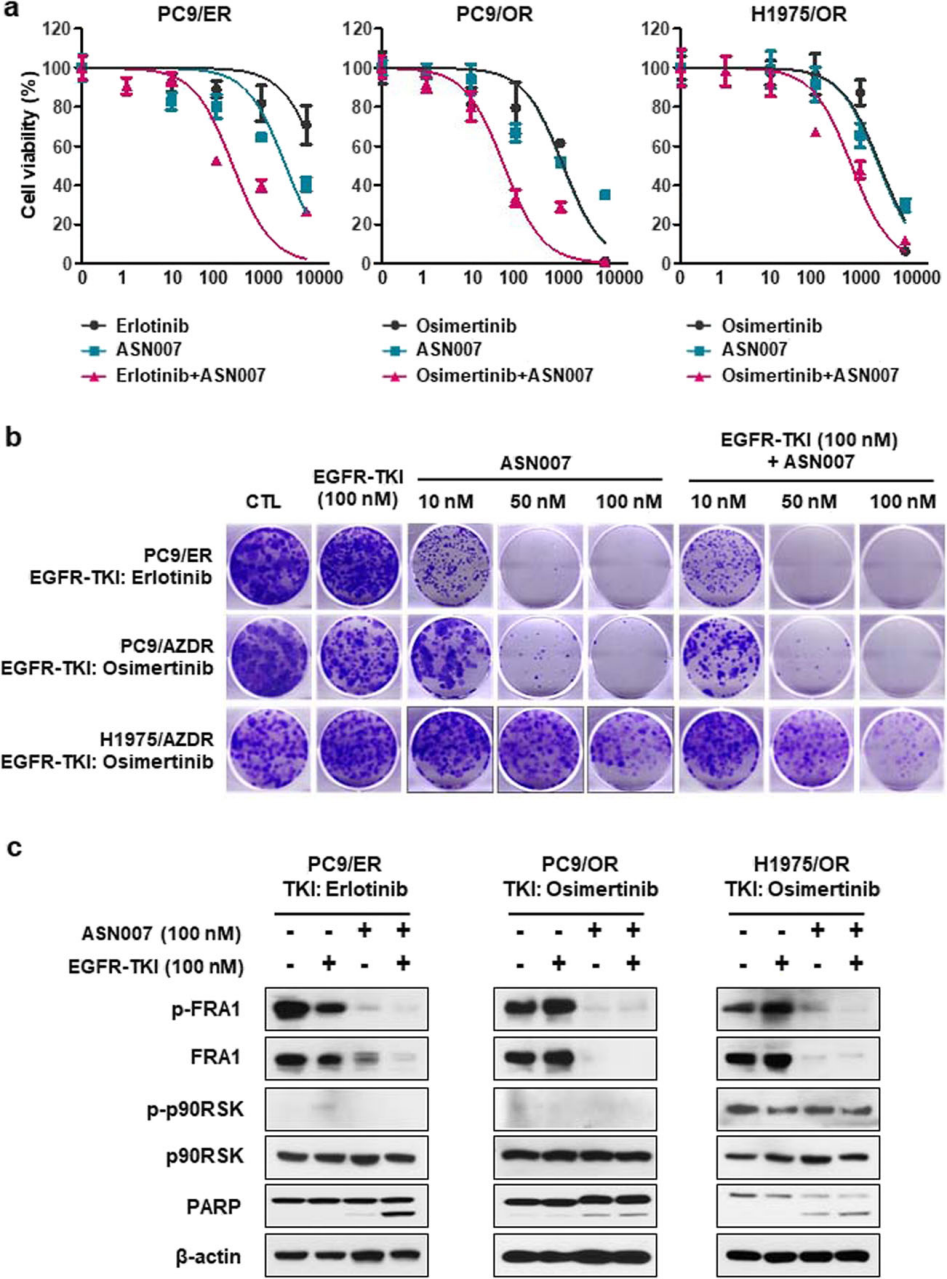
ERK-dependent resistance to EGFR TKI is overcome by combination treatment with ASN007. **a** Cell viability after EGFR TKI alone, ASN007 alone, or combination treatment for 72 h. Data are presented as mean ± SEM (n = 6). **b** Colony formation after EGFR TKI and ASN007 combination treatment for 10–14 days. **c** Western blot analysis showing FRA1, p90RSK, and PARP levels after 24-h treatment with EGFR TKI alone, ASN007 alone, and a combination of EGFR TKI and ASN007. β-actin was used as a loading control

Based on these findings, we tested whether ASN007 could overcome the acquired resistance to EGFR TKIs. Combination treatment with EGFR TKIs and ASN007 was more effective than either single agent alone with regard to short-term cell viability in PC9/ER, PC9/OR, and H1975/OR cells (Fig. 4a). Furthermore, ASN007 alone as well as co-treatment with EGFR TKI reduced the cell viability for long-term (10–14 days) incubation (Fig. 4b). The ASN007 alone or combination with EGFR TKI markedly inhibited the phosphorylation of FRA1 and induced PARP cleavage compared with EGFR TKI alone (Fig. 4c).

### Combination treatment with erlotinib and ASN007 effectively inhibits the growth of PC9/ER xenografts *in vivo*

**Fig. 5:**
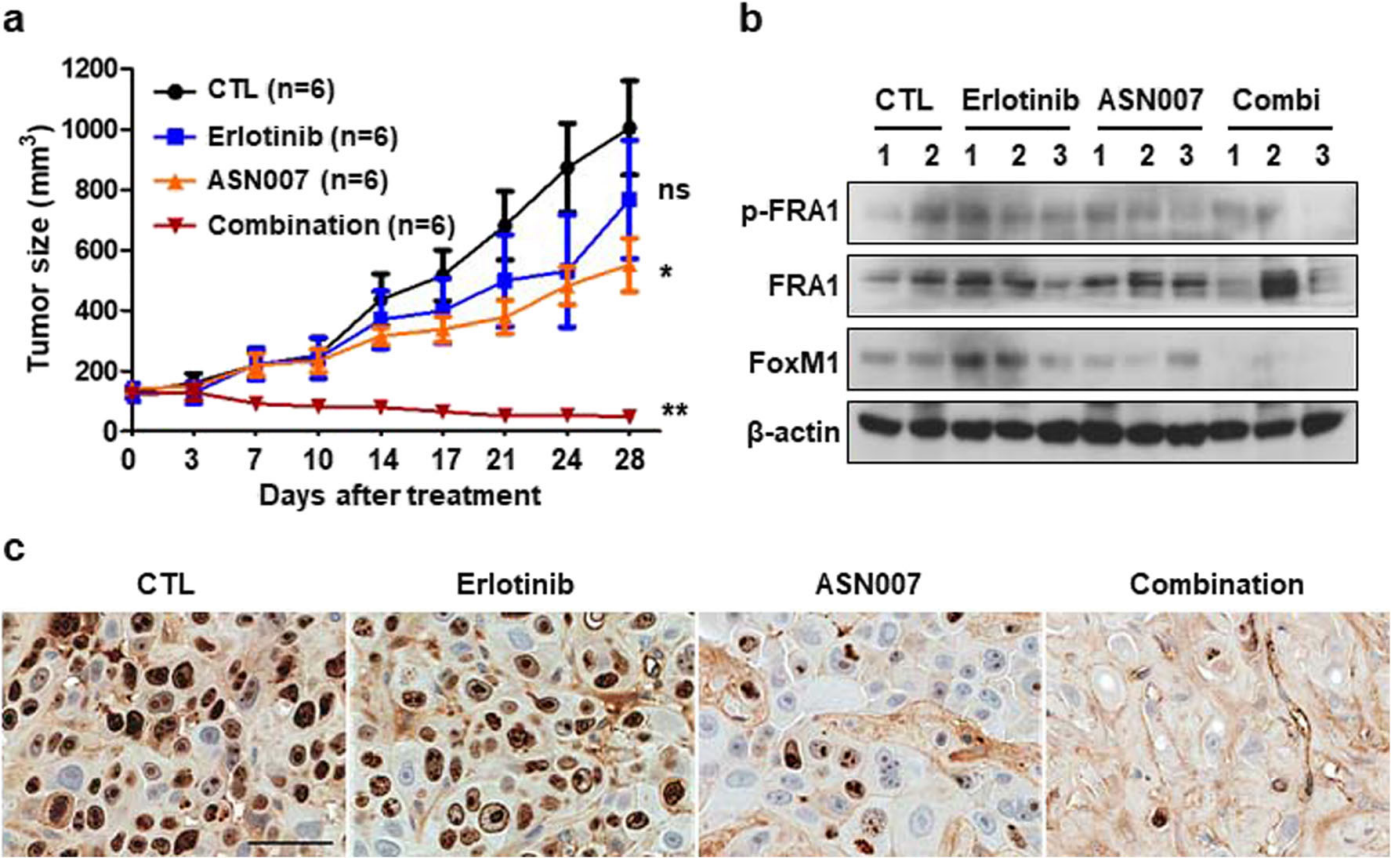
The combination of ASN007 with erlotinib effectively inhibits the growth of erlotinib-resistant xenografts. **a** Tumor growth of PC9/ER xenograft treated with vehicle, erlotinib (25 mg/kg/d), ASN007 (50 mg/kg/d), and a combination of erlotinib and ASN007 by oral gavage for 5 days each week. **b** Western blot analysis of FRA1 and FoxM1 in tumor samples treated as indicated. β-actin was used as a loading control. **c** Immunohistochemical analysis using Ki-67. Scale bar: 50 μm. *, *P* < 0.05; **, *P* < 0.01

To determine whether ASN007 could overcome EGFR TKI resistance *in vivo*, we used PC9/ER xenograft because the majority of patients in this study were treated with first-generation EGFR TKIs. Xenograft tumors induced by PC9/ER cells continued to grow *in vivo* with or without erlotinib treatment for 28 days, indicating erlotinib resistance. ASN007 alone significantly decreased tumor growth and the combination of ASN007 with erlotinib completely inhibited tumor growth in PC9/ER xenografts (Fig. 5a). Consistent with growth inhibition, ASN007 treatment in combined with erlotinib showed further inhibition of FRA1 phosphorylation and FoxM1 expression compared to ASN007 alone (Fig. 5b). In addition, cell proliferation was synergistically inhibited by combination therapy with erlotinib and ASN007, as assessed by Ki-67 expression (Fig. 5c). These findings confirm the *in vitro* results and support the role of ERK signaling in promoting acquired resistance to EGFR TKI.

## Discussion

Several studies have shown that NSCLC cells treated with EGFR TKIs adopt multiple mechanisms to reactivate ERK signaling. The upregulation of phosphorylated ERK following EGFR TKI resistance was a ubiquitous event in EGFR TKI-resistant NSCLC tissues and cell lines. Our study suggested that targeting ERK is an effective strategy to overcome acquired resistance to EGFR TKIs in NSCLC. Furthermore, co-targeting EGFR and ERK will be an even more effective combination strategy.

ERK (ERK1/2) controls several downstream cytoplasmic and nuclear targets by phosphorylating and regulating cell cycle and negative feedback mechanisms [15]. Although specific ERK alterations have not been identified as actionable mechanisms of acquired resistance to TKIs, alterations in upstream of ERK are common in acquired resistance to various TKIs including EGFR TKI [1, 18]. Previous studies reported that acquired resistance to EGFR TKIs converged on the activation of the MAPK pathway, especially ERK, albeit through different mechanisms such as MET amplification and RAS alteration [2, 9, 19]. Recently, reactivation of ERK signaling through chemokine receptor CXCR7 has been identified as a resistance mechanism to EGFR TKI in patients with NSCLC [3]. Therefore, targeting ERK would be an attractive strategy for the treatment of a variety of tumor types harboring acquired resistance to TKIs.

Several ERK inhibitors including ASN007, ulixertinib, and LY3214996 are being developed in clinical trials as a treatment for advanced solid tumors with RAS-RAF-MAPK pathway alterations [15]. Some have demonstrated preliminary antitumor activity in preclinical and clinical trials. However, the most common or dose-limiting toxicities observed to date with ERK inhibitors include diarrhea, nausea, fatigue, and rash. Given that ASN007 is a highly potent and selective ERK1/2 inhibitor in the nanomolar range with a long target residence time, it can be hypothesized that toxicities related to AN007 might be reduced compared to other ERK inhibitors. In our study, ASN007 shows promising antitumor activity as a single agent and in combination with EGFR TKI. ASN007 alone did not cause any dose-limiting toxicities including loss of body weight or skin rash in a xenograft model. Although mice treated with a combination of ASN007 and erlotinib showed body weight loss 1 week after treatment, this loss was recovered after 2 weeks of treatment. These observations suggest that our proposed combination of ASN007 plus EGFR TKI was well tolerated in a mouse xenograft model.

Intriguingly, we found that decreased FRA1 expression is the main mechanism of ERK inhibition by ASN007 in EGFR TKI-resistant NSCLC. In contrast, previous studies using other ERK inhibitors such as ulixertinib and LY3214996 demonstrated that the main downstream target of ERK in *KRAS*-driven tumors is RSK, not FRA1 [20, 21]. Although phosphorylation of RSK was also inhibited by ASN007 treatment, FRA1 protein expression is more abundant than that of RSK1 in our resistant cells. These results suggest that ERK inhibitor could exert antitumor effects via different mechanisms according to tumor context. FRA1 is a member of the FOS protein family and can form an AP-1 transcription factor. FRA1 is mainly regulated by post-translational phosphorylation by a mitogen-activated protein kinase (MAPK) signaling pathway, especially ERK. Because phosphorylation of FRA1 prevents degradation by ubiquitin-independent proteasome, ERK activation is required for FRA1 accumulation. Many studies have shown that FRA1 is overexpressed in many tumors such as lung cancer, breast cancer, colorectal cancer and other tumors. The abnormal expression of FRA1 in tumor has important roles during tumor progression, promoting cell proliferation and invasion, inhibiting apoptosis, and enhancing tumor angiogenesis and heterogeneity [16, 22, 23]. Previous studies reported that FRA1 promotes KRAS-induced lung cancer progression and metastasis [17, 24, 25]. In addition, FRA1 contributed to oncogenic KRAS-driven PD-L1 expression in high risk, premalignant human bronchial epithelial cells, suggesting that FRA1 may promote cancer progression by facilitating immune evasion [14]. Altogether, FRA1 may be a prognostic marker and potential target for lung cancer with oncogenic mutations or drug resistance.

Our study has several limitations. First, the EGFR TKI resistance models in this study may not represent the variety of clinical situations in patients with EGFR TKI resistance observed in clinic. Although *in vitro* models we developed in this study have different genomic alterations in upstream of ERK as resistance mechanism, it converged ERK activation regardless of EGFR TKIs. Second, the regulation of acquired resistance by ERK has not been fully elucidated. Further mechanistic studies are needed to investigate how increased ERK signaling exclusively activates the development of drug resistance.

In summary, our findings suggest that concomitant EGFR and ERK blockade is a promising strategy to overcome acquired resistance in *EGFR*-mutated NSCLC regardless of whether the acquired resistance arises from first- or third-generation EGFR TKIs. Further research is needed to determine whether these combinations can also prevent or delay the development of acquired resistance.

## Data Availability

The data and material generated and analyzed during the present study are available from the corresponding author on reasonable request.
